# *In situ* and real-time monitoring of structure formation during non-reactive sputter deposition of lanthanum and reactive sputter deposition of lanthanum nitride

**DOI:** 10.1107/S1600576718007367

**Published:** 2018-06-28

**Authors:** Bärbel Krause, Dmitry S. Kuznetsov, Andrey E. Yakshin, Shyjumon Ibrahimkutty, Tilo Baumbach, Fred Bijkerk

**Affiliations:** a Institut für Photonenforschung und Synchrotronstrahlung (IPS), Karlsruher Institut für Technologie (KIT), Karlsruhe, Germany; b Industrial Focus Group XUV Optics, MESA+ Institute for Nanotechnology, University of Twente, Enschede, The Netherlands; c Max Planck Institute for Solid State Physics, Stuttgart, Germany; d Laboratorium für Applikationen der Synchrotronstrahlung (LAS), Karlsruher Institut für Technologie (KIT), Karlsruhe, Germany

**Keywords:** sputter deposition, nitrides, X-ray optics, extreme UV

## Abstract

Lanthanum and lanthanum nitride thin films were deposited by magnetron sputtering onto silicon wafers covered by natural oxide. *In situ* and real-time synchrotron radiation experiments during deposition reveal that lanthanum crystallizes in the face-centred cubic bulk phase. Lanthanum nitride, however, does not form the expected NaCl structure but crystallizes in the theoretically predicted metastable wurtzite and zincblende phases, whereas post-growth nitridation results in zincblende LaN. During deposition of the initial 2–3 nm, amorphous or disordered films with very small crystallites form, while the surface becomes smoother. At larger thicknesses, the La and LaN crystallites are preferentially oriented with the close-packed lattice planes parallel to the substrate surface. For LaN, the onset of texture formation coincides with a sudden increase in roughness. For La, the smoothing process continues even during crystal formation, up to a thickness of about 6 nm. This different growth behaviour is probably related to the lower mobility of the nitride compared with the metal. It is likely that the characteristic void structure of nitride thin films, and the similarity between the crystal structures of wurtzite LaN and La_2_O_3_, evoke the different degradation behaviours of La/B and LaN/B multilayer mirrors for off-normal incidence at 6.*x* nm wavelength.

## Abstract 2

A real-time synchrotron radiation study of the crystalline phase, texture formation and resulting surface roughness during deposition of thin La and LaN films is presented. For LaN, the theoretically predicted metastable wurtzite and zincblende structures were found, while La assumes the expected NaCl structure.

## 1. Introduction   

Multilayer (ML) mirrors in the soft X-ray and extreme ultraviolet (EUV) regimes are employed for various applications, including EUV telescopes for space research (Culhane *et al.*, 2007 ▸; Lemen *et al.*, 2012 ▸), beam-transport systems and focusing optics for free-electron lasers (Corso *et al.*, 2012 ▸; Nelson *et al.*, 2009 ▸), EUV lithography (Louis *et al.*, 2011 ▸) and total-reflection X-ray fluorescence analysis (TXRF) (Tiwari *et al.*, 2010 ▸).

Lanthanum and boron are very promising candidates for mirror applications focusing on wavelengths of around 6.*x* nm (Hawryluk & Ceglio, 1993 ▸; Makhotkin *et al.*, 2012 ▸). Experimentally, a reflectance of 64.1% for near-normal incidence was obtained by a hybrid process combining non-reactive and reactive sputter deposition of La. Delayed nitridation of the La layer avoids the formation of undesired BN at the La-on-B interface, while a fully passivated LaN layer on top significantly reduces the B-on-La interface width (Makhotkin *et al.*, 2013 ▸; Kuznetsov *et al.*, 2015 ▸, 2016*a* ▸).

For application purposes, the long-term stability of the multilayers is crucial. Lanthanum and lanthanum nitride are highly reactive under ambient conditions, while crystalline and amorphous boron particles are known to oxidize only within a limited thickness in the range 0.5–2 nm (Van Devener *et al.*, 2009 ▸; Jain *et al.*, 2010 ▸; Shin *et al.*, 2011 ▸). Therefore, a boron cap thicker than about 2–3 nm is expected to protect La/B and LaN/B multilayers from oxidation. Surprisingly, this approach works for normal-incidence mirrors (ML periods of around 3.5 nm) but not for grazing-incidence mirrors with larger thickness: while La/B structures with an ML period of 15 nm appeared to be stable to storage for at least six months, LaN/B multilayers with the same period showed strong surface degradation after one week of storage in air (Kuznetsov *et al.*, 2016*b* ▸). This thickness-dependent degradation behaviour suggests that the stability of the cap might be influenced by the properties of the underlying layer. The main objective of this manuscript is to determine the crystalline phases and microstructures of La and LaN thin films.

Only limited information is available on the local atomic ordering and microstructures of La and LaN thin films and nanostructures (Nyabero *et al.*, 2014 ▸; Kuznetsov *et al.*, 2016*a* ▸; Zhang *et al.*, 2017 ▸). For bulk lanthanum, the low-temperature double hexagonal close-packed α phase (Inorganic Crystal Structure Database entry ICSD-641382; *a* = 3.772, *c* = 12.144 Å; Beaudry & Palmer, 1974 ▸) with a phase transition to a high-temperature face-centred cubic (f.c.c.) β phase (ICSD-41518; *a* = 5.32 Å; Haglund *et al.*, 1993 ▸) at 566 ± 10 K was reported (Konings & Beneš, 2010 ▸). For lanthanum nitride, the NaCl structure with *a* = 5.293 Å (ICSD-641470; Ettmayer *et al.*, 1980 ▸) was reported. However, *ab initio* calculations predict the formation of other metastable phases at room temperature: a wurtzite (WZ) phase with *a* = 3.95–4.2 Å and *c* = 5.84–6.24 Å, and a zincblende (ZB) phase with *a* = 5.51–5.69 Å (Zhao & Wu, 2008 ▸; Ghezali *et al.*, 2008 ▸).

Owing to the high reactivity of La and LaN, the crystal phases and microstructures of these materials are difficult to access. This problem can only be overcome by *in situ* analysis methods, avoiding any contact of the thin films with the highly reactive contaminants oxygen or nitrogen, with humidity, or with other surface contaminants such as hydrocarbons. In this paper, we report on the structural evolution of La and LaN single layers during thin-film formation. The chemical composition and bond structure of the layers were verified by X-ray photoelectron spectroscopy (XPS) measurements. Reflection high-energy electron diffraction (RHEED) measurements gave a first indication of the crystalline phases. The reactive and non-reactive sputter processes of lanthanum, and the post-growth nitridation of a lanthanum layer, were monitored by combined *in situ* and real-time synchrotron X-ray diffraction (XRD) and X-ray reflectivity (XRR) measurements (Kaufholz *et al.*, 2015 ▸; Krause *et al.*, 2016 ▸). The phase formation and texture development could be determined and related to the simultaneously detected film thickness and roughness. This detailed understanding of structure formation during La and LaN thin-film deposition is required for a deeper insight into the structural details and long-term stability of 6.*x* nm multilayer stacks, for both normal and grazing incidence.

## 2. Experimental   

### 2.1. Thin-film deposition   

The La and LaN layers were deposited in a portable magnetron sputtering chamber dedicated to *in situ* X-ray experiments during reactive and non-reactive magnetron sputtering (Krause *et al.*, 2012 ▸). The base pressure of the ultra-high-vacuum (UHV) system was 1 × 10^−6^ Pa. The lanthanum target, with a diameter of 50 mm, was mounted at a distance of about 130 mm from the earthed 20 × 20 × 1 mm Si substrate. All substrates used for the *in situ* X-ray experiments were taken from the same wafer. Sample exchange was done using a loadlock, reducing the contamination of the La target to a minimum.

Before each deposition, the target was sputter-cleaned. During this period, the substrate was protected by a shutter. Non-reactive sputter deposition was done with a flux of 1.2 sccm (standard cubic centimetres per minute) argon, corresponding to a pressure of about 0.3 Pa. During reactive sputter deposition, a mixture of 0.6 sccm Ar and 1.2 sccm N_2_ at a pressure of 0.55 Pa was used. The DC power at the La target was kept at 20 W, resulting in a target voltage of 267 V during La deposition and 188 V during deposition of LaN. A similar voltage difference between reactive and non-reactive La sputtering was also observed by Haye *et al.* (2016 ▸).

For the RHEED and XPS measurements, the sputter chamber was docked to a UHV cluster system with a base pressure of 1 × 10^−8^ Pa. The samples were transferred to the analysis chambers directly after growth, without exposure to ambient conditions. The XPS measurements were performed with a Phoibos 150 analyser and an unmonochromated XR-50 Mg *K*α X-ray source from SPECS which was calibrated using the Ag 3*d* 5/2 peak. For the LaN coatings, a floodgun was used for charge compensation. The RHEED measurements were carried out at an electron energy of 10 keV, using an REG30 electron gun (Dr Gassler Electronic Devices).

**Figure 1:**
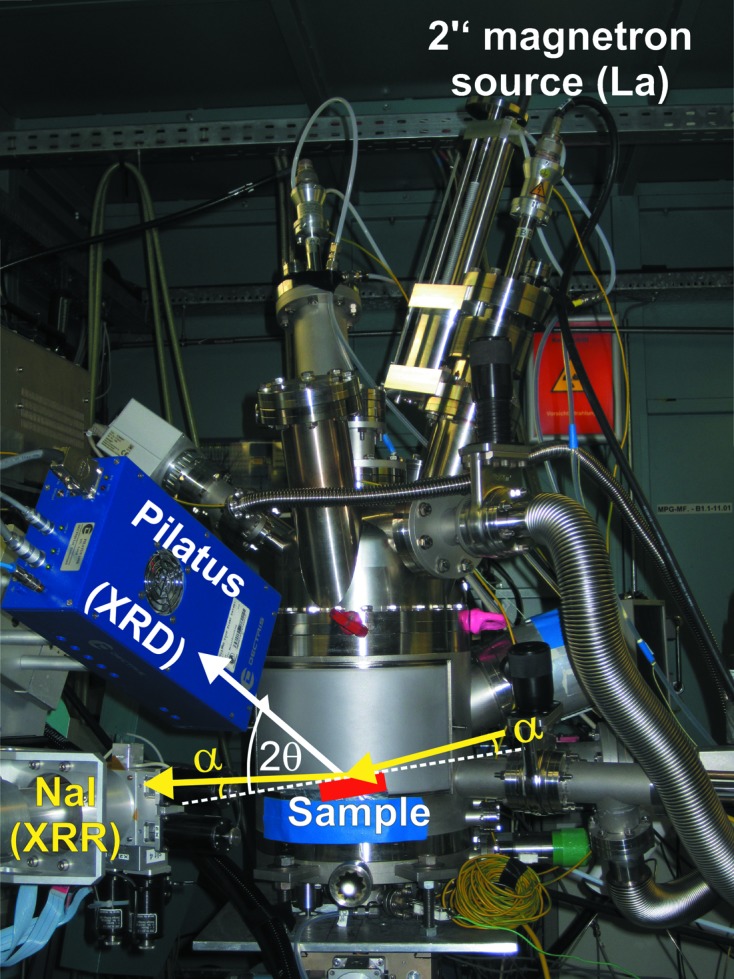
Experimental setup and schematic of the measurement geometry. The sample horizontal is indicated as a dashed line. The incoming beam at an incident angle α is reflected onto a point detector (yellow arrows), while the diffracted signal (white arrow) is measured with a two-dimensional detector.

The XRD and XRR experiments were performed at the MPI beamline of the Test Facility and Synchrotron Radiation Source ANKA, Karlsruhe, Germany (Stierle *et al.*, 2004 ▸). The experimental setup and measurement geometry are shown in Fig. 1 ▸. The sputter chamber was mounted in horizontal scattering geometry on a Huber 4 + 2 heavy-load diffractometer. Two detectors, an NaI scintillation detector and a Pilatus 100K detector (Dectris), were mounted with an angular offset of 24° on the same detector arm. For the time-dependent X-ray experiments during thin-film deposition, the incoming X-ray beam with a photon energy of 10 keV, an incident angle α = 1.5° and a beam size of 0.3 × 0.15 mm (horizontal × vertical) was reflected onto the scintillation detector. Simultaneously, the diffracted signal was measured with a two-dimensional detector, mounted at 2θ = 27° and covering an angular range of about ±6° in the vertical direction and ±2° in the horizontal direction. Both data sets were recorded with a sampling frequency of 1 s. Before and after deposition, angular reflectivity scans and two-dimensional maps of the diffraction signal were measured. For the maps, the Pilatus detector was scanned in an angular range of ±30° in the horizontal direction and 0–40° in the vertical direction. The angular scans were done with automatic absorbers. The measured signal was normalized to the incoming beam intensity, which was monitored by an ionization chamber. The reproducibility of all observations was verified by the repeated deposition of films using identical growth conditions.

## 3. Results   

### 3.1. Surface analysis   

**Figure 2:**
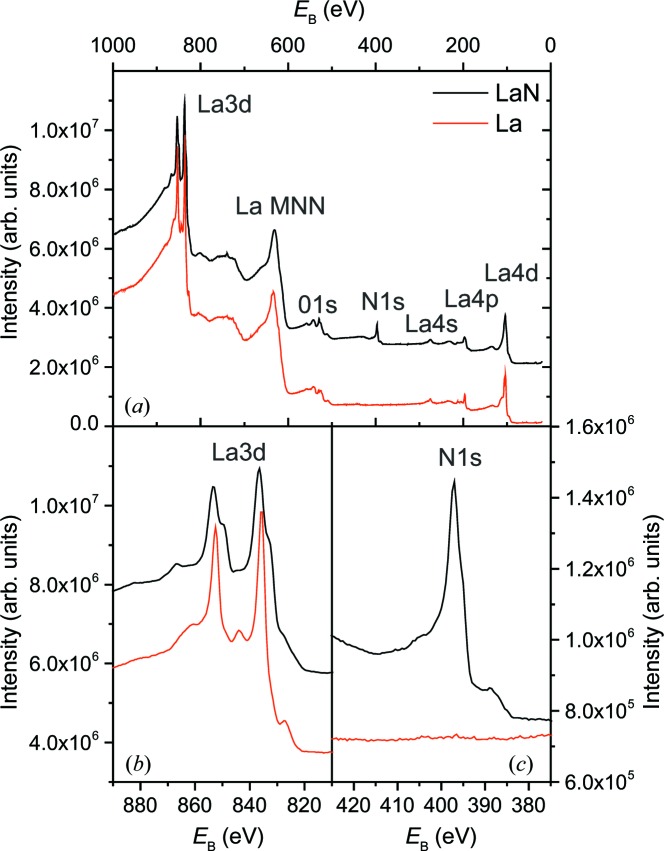
XPS spectra after non-reactive (red lines) and reactive (black lines) sputter deposition. (*a*) Survey spectra, (*b*) an enlargement around La 3*d*, and (*c*) an enlargement around N 1*s*.

To verify the chemical composition and bond structure, XPS measurements were performed on thin films deposited under non-reactive and reactive sputter conditions. The results are shown in Fig. 2 ▸. For the reactively sputtered LaN film (black lines), the overview spectrum shows the lanthanum XPS peaks La 3*d*, La 4*s*, La 4*p* and La 4*d*, and the nitrogen peak N 1*s* (Fig. 2 ▸ *a*). With the exception of the nitrogen peak, the same features are also observed for non-reactive sputter deposition of lanthanum (red lines). For both materials, an oxygen content of about 5 at.% was found at the sample surface, but quantification is difficult because the O 1*s* peak is superimposed on the strong La Auger signal.

Bond formation is revealed by an enlargement of the La 3*d* region (Fig. 2 ▸ *b*). For non-reactive deposition, two well separated narrow peaks at binding energies of 835.7 ± 0.2 (La 3*d* 5/2) and 852.5 ± 0.2 eV (La 3*d* 3/2) are observed, as expected for metallic La (Kumar *et al.*, 1984 ▸). For reactive sputtering both peaks showed a double-peak structure. The La 3*d* 5/2 peak is shifted to 333.3 ± 0.3 eV, which is lower than for metallic La, and a satellite peak with higher intensity occurs at 836.6 ± 0.2 eV. This satellite structure is a fingerprint of the chemical environment of La: La(OH)_3_ formation leads to a smaller satellite peak, while La_2_O_3_ is characterized by two peaks of similar height (Sunding *et al.*, 2011 ▸). The satellite peak with higher intensity observed here is characteristic of LaN (Coss *et al.*, 2009 ▸; Kuznetsov *et al.*, 2016*a* ▸). The La—N bond is also confirmed by the N 1*s* peak at 397 eV, which is typical for metal–nitrogen bonds.

**Figure 3:**
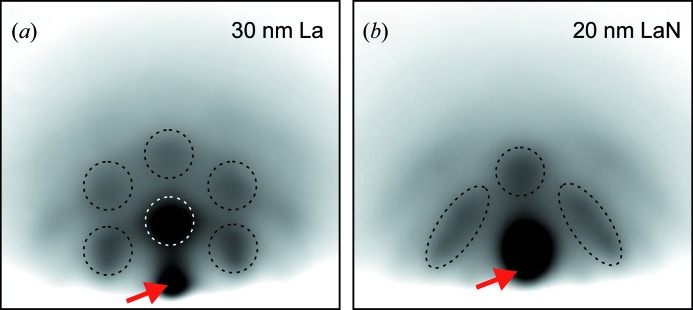
RHEED images of film growth by (*a*) non-reactive and (*b*) reactive deposition from a lanthanum target. The red arrows indicate the reflected electron beam. Characteristic diffraction spots are highlighted by dashed lines.

The difference between reactively and non-reactively deposited films was also confirmed by RHEED measurements (see Fig. 3 ▸). For both film types, spotty RHEED patterns typical of the transmission of an electron beam through three-dimensional structures were observed. The non-reactively deposited La film shows the hexagonal pattern expected for f.c.c. La in the [111] orientation. For reactive deposition, a different pattern with more pronounced intensity wings is found, which cannot be explained by LaN in the expected NaCl structure. Similar patterns are also observed for the deposition of La and LaN on boron, *i.e.* phase formation on Si wafers is also relevant for La/B-based multilayers.

### 3.2. Crystalline phases   

**Figure 4:**
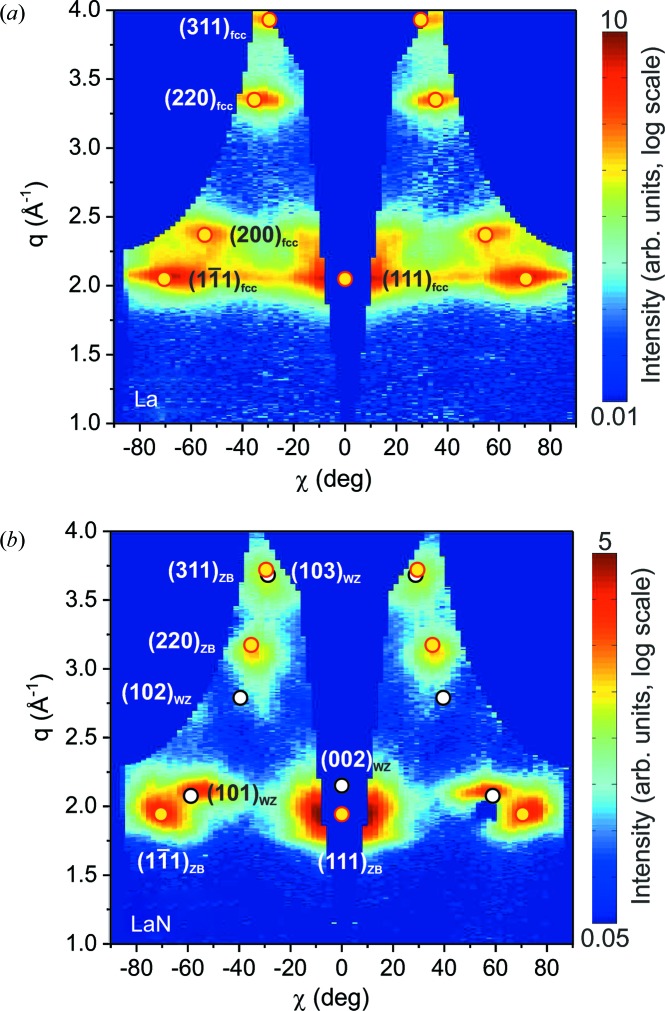
Diffraction maps after (*a*) non-reactive and (*b*) reactive deposition, plotted as a function of the momentum transfer *q* and the angle χ to the surface normal. The non-reactive deposition results in a [111] textured f.c.c. La film. Yellow dots correspond to the expected peak positions. The pattern observed after reactive deposition can be explained by coexisting wurtzite and zincblende LaN in [002] and [111] orientations, respectively. The expected zincblende (yellow) and wurtzite (white) peak positions are indicated.

*In situ* synchrotron studies of the X-ray diffraction signal reveal a more detailed picture of the crystalline structure. Fig. 4 ▸ shows X-ray diffraction maps of (*a*) La and (*b*) LaN films with a thickness of 30 nm, measured directly after deposition while keeping the films under UHV conditions. The data are presented as *q*/χ maps, where χ is the angle to the surface normal and *q* is the scattering momentum transfer in a radial direction (corresponding to a 2θ/ω scan). In this representation, a perfect stress-free crystalline powder would be characterized by horizontal lines representing the diffraction rings.

The La pattern (Fig. 4 ▸ *a*) corresponds well to the f.c.c. [111] texture of the lanthanum β phase (indicated by yellow dots). The weaker intensity distribution above the central (111) peak indicates a coexisting [100] texture. Assuming thermodynamic equilibrium, the lanthanum β phase is expected at temperatures above ∼566 K, which is well above our nominal substrate temperature of about 300 K during deposition. This can be explained in two ways: (i) for thin films the transition temperature might be shifted because of the energetic contributions of the surface and interface to the substrate; and (ii) the non-equilibrium sputter deposition process, where energetic particles are deposited on the growing film, often results in the growth of metastable phases. Once the β phase is formed, it is likely to be stabilized at room temperature owing to the extremely slow kinetics of the β-to-α phase transition (Konings & Beneš, 2010 ▸).

The LaN pattern shown in Fig. 4 ▸(*b*) shows a much shorter distance between the two side peaks close to *q* = 2 Å^−1^ and χ = 70°. The pattern cannot be explained by the expected NaCl structure, but agrees well with the theoretically predicted wurtzite and zincblende structures (Zhao & Wu, 2008 ▸; Ghezali *et al.*, 2008 ▸). Fig. 4 ▸(*b*) shows the calculated peak positions for [111]-oriented ZB LaN with *a* = 5.6 Å (yellow dots) and [002]-oriented WZ LaN with *a* = 4.08 Å and *c* = 5.84 Å (white dots). The observed structures are very interesting from the electronic point of view: while the expected NaCl structure is predicted to be metallic or semi-metallic, the WZ and ZB structures are expected to be semiconductors (Ghezali *et al.*, 2008 ▸).

**Figure 5:**
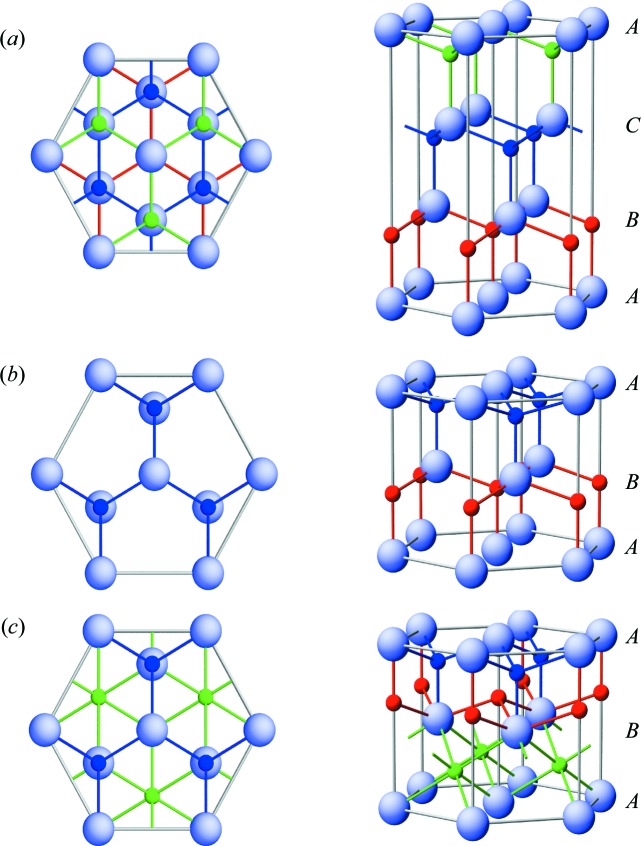
Layer stacking of (*a*) the zincblende structure, (*b*) the wurtzite structure and (*c*) La_2_O_3_. The left-hand column shows top views and the right-hand column side views of the close-packed structures. Large light-blue balls correspond to lanthanum atoms and small balls to nitrogen or oxygen atoms. For better visibility, different colours indicate lattice sites in different atomic planes along the stacking direction.

The ZB structure has *ABC* layer stacking and the WZ structure *AB* layer stacking, as shown in Figs. 5 ▸(*a*) and 5 ▸(*b*), respectively. Both structures are close packed and directly related by stacking faults. A similar polytypism has been reported, for example, for GaN (Paulus *et al.*, 1997 ▸), GaAs (Schroth *et al.*, 2015 ▸) and BN (Kester *et al.*, 1993 ▸). For electronic applications, coexisting phases are often undesired, while for hard coating materials such as BN, coexisting structures offer new alternatives to tune the material properties. Interestingly, the wurtzite structure of LaN has the same metal sublattice as La_2_O_3_ (Fig. 5 ▸ *c*). It is possible that small oxygen impurities stabilize the metastable wurtzite structure, as known, for example, for the metastable tungesten β phase (Demasius *et al.*, 2016 ▸). The similarity of the two crystalline lattices might also be relevant for the degradation mechanism of LaN under ambient conditions.

### 3.3. Structure formation during reactive and non-reactive deposition   

The LaN layer thickness has a strong influence on the degradation behaviour of boron-capped LaN, while boron-capped La layers seem to be stable in the studied La thickness range of up to 15 nm (Kuznetsov *et al.*, 2016*b* ▸). Therefore, the thickness dependence of the structure formation was studied during thin-film deposition, employing a combination of *in situ* XRD and XRR. This approach has already been used successfully to understand the structure formation of MoSi_*x*_/Si layer systems (Krause *et al.*, 2016 ▸).

**Figure 6:**
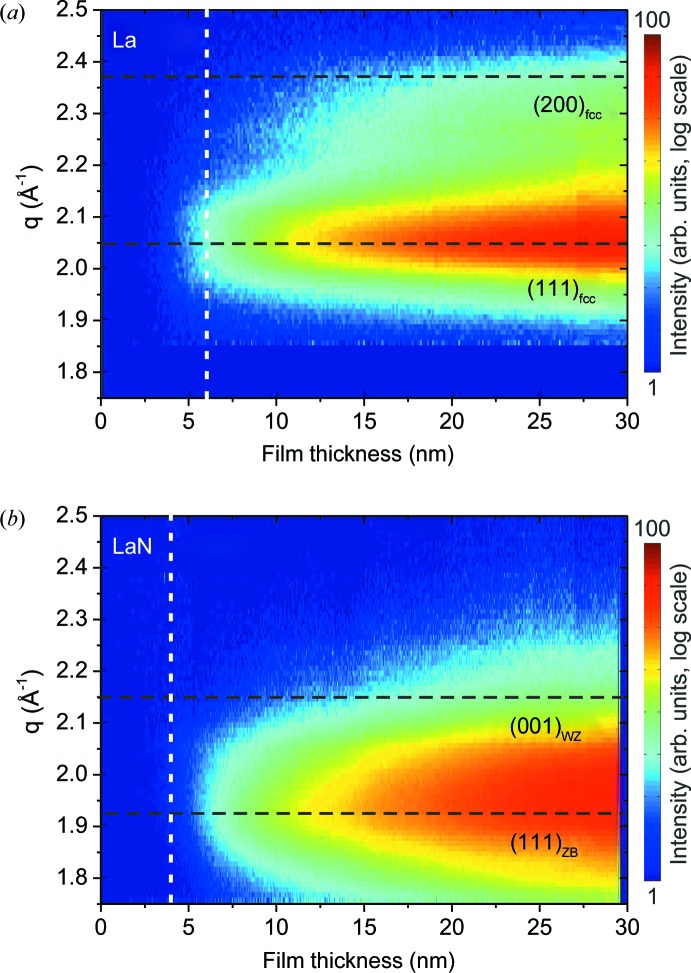
X-ray diffraction signals during (*a*) non-reactive and (*b*) reactive deposition, plotted as a function of film thickness. The nominal peak positions of the crystalline phases identified after growth are indicated by black dashed lines. The dashed white lines (vertical) indicate the film thickness with minimum roughness.

Fig. 6 ▸ shows the time-dependent diffraction signals close to the surface normal of the substrate (χ ≃ 14°), measured for (*a*) non-reactive and (*b*) reactive deposition. For better comparison, the deposition time has been converted into film thickness. The deposition rate was determined from the time-dependent XRR signal (see below). At thicknesses lower than 2–3 nm, both films show a weak and broad intensity distribution, indicating an amorphous layer or small disordered crystallites. During further deposition, in both cases the final texture is established within a few nanometres. For non-reactive deposition, an intense La(111) and a weak La(200) peak emerge. For reactive deposition, the intensity distribution remains broad but becomes slightly asymmetric, confirming the coexistence of LaN WZ and ZB phases already during early growth.

**Figure 7:**
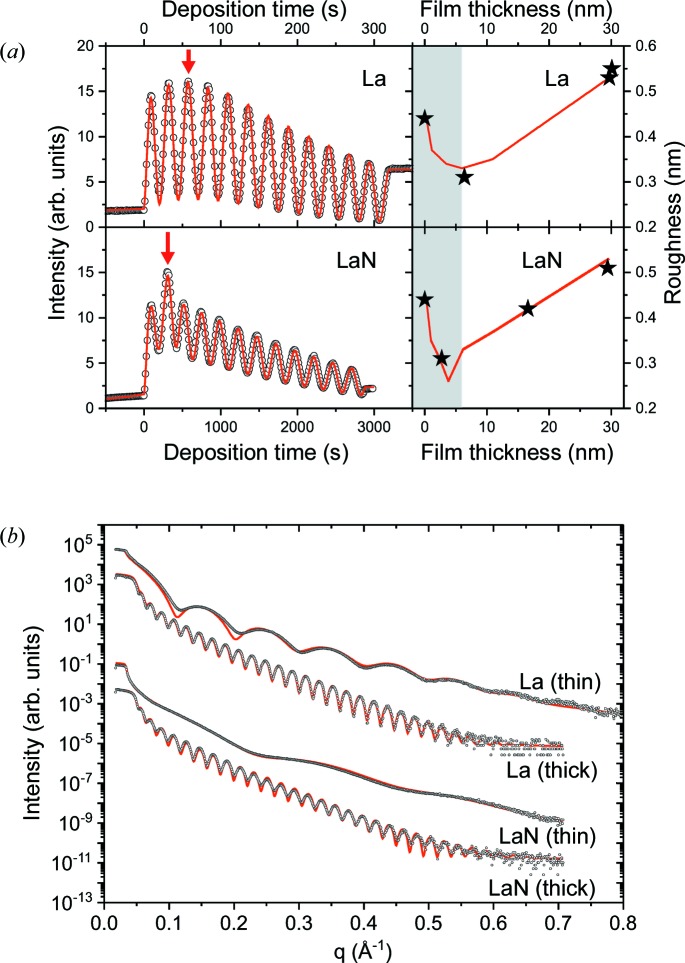
Experimental (open symbols) and simulated (red line) XRR measurements, (*a*) during and (*b*) after non-reactive and reactive deposition. The time-dependent measurements were performed at *q* = 0.2653 Å^−1^. The roughness development with film thickness (extracted from the fit, red line) is shown on the right-hand side. Reference values determined from fitting complete angular XRR measurements at the indicated thickness are shown as black stars, and the initial growth regime is highlighted (grey background). The angular measurements were performed on thin and thick (∼30 nm) La and LaN films. For the thin films, the deposition was interrupted close to the maximum intensity of the time-dependent measurements [red arrow in panel (*a*)]. The fit parameters are summarized in Table 1 ▸.

The time-dependent reflectivity signal, measured during non-reactive and reactive deposition, is presented in Fig. 7 ▸(*a*). The intensity was collected simultaneously with the XRD signal shown in Fig. 6 ▸. The experimental data are indicated by open symbols. They show characteristic oscillations which reflect the increasing film thickness. During one oscillation period τ, a film of thickness
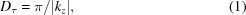
is deposited, where |*k_z_*| is the *z* component of the wavevector *k* = 2π/λ. At the incident angle α = 1.5° (*q* = 0.2653 Å^−1^), each oscillation corresponds to the deposition of approximately 2.40 nm.

The time-dependent experimental data were fitted using the Parratt formalism, as described in detail by Kaufholz *et al.* (2015 ▸) and Krause *et al.* (2016 ▸). First, the angular reflectivity scans before and after deposition were fitted assuming a simple layer model. This was done for thick films of ∼30 nm, and thin films where the deposition was interrupted close to the maximum of the time-dependent XRR signal. Based on these reference values, a time-dependent layer model was developed. The experimental data were reproduced by optimization of the thickness increase and roughness change during deposition. The fitted angular and time-dependent curves are shown as red lines in Fig. 7 ▸.

Table 1 ▸ summarizes the simulation parameters of the angular reflectivity curves. For the thick LaN film, a two-layer model was assumed. The other measurements could be reproduced with a single layer. The fit parameters for the substrate, consisting of silicon covered by a natural oxide layer, were the same for all samples.

The expected δ values are δ(La) = 10.49 × 10^−6^ for f.c.c. and h.c.p. La, δ(f.c.c. LaN) = 11.81 × 10^−6^ for LaN with the NaCl structure, and δ(WZ/ZB) = 9.6–10.3 × 10^−6^ predicted for WZ and ZB LaN. For La, the measured value is only slightly lower than expected. For LaN, the values are too low for the NaCl structure but can be explained by WZ and ZB LaN, in agreement with the diffraction data. For a pure LaN layer, the δ value is proportional to the mass density of the film. The slightly lower δ value of the LaN layer during the first few nanometres can be explained by porosity. Assuming that the subsequently deposited LaN (layer 2) has bulk density, the density of the first layer would be in the range of ∼80% (thin film) to ∼90% (thick film). However, owing to the low inter­action with the SiO_*x*_ surface compared with B, it is not clear if this observation is relevant for multilayer formation. In agreement with our XPS measurements, a large oxide contribution can be excluded since all observed values are much lower than δ(La_2_O_3_) = 11.68 × 10^−6^.

From the fit of the time-dependent curves, deposition rates of 0.091 nm s^−1^ for non-reactive and 0.010 nm s^−1^ for reactive deposition were obtained. For both layers, an initial smoothing of the films was found, consistent with the fit parameters obtained for the angular XRR of the thin films. For LaN, the minimum roughness is reached at about 3 nm film thickness, and there is subsequently a continuous roughness increase which can be explained by simultaneous texture formation. For La, the behaviour is similar but the roughness minimum is broader and shifted to a larger film thickness of 5–6 nm, which is slightly delayed with respect to the onset of texture formation.

Texture formation is often accompanied by facet formation. The surface morphology coarsens with increasing thickness, resulting in an increasing roughness, as observed for LaN and La. However, the detailed growth mechanisms of the two mater­ials are different, as indicated by the delayed roughness transition observed for La. This delayed transition can be explained by grain-boundary diffusion taking place in competition with vertical grain growth. Because of this mechanism, the gaps between neighbouring grains are filled, leading to an additional smoothing effect (Chason, 2012 ▸). Nitrides have a lower mobility than metals. After the onset of faceting, shadowing effects suppress the formation of grain boundaries, and a columnar film with small voids is formed (Petrov *et al.*, 2003 ▸; Nita *et al.*, 2016 ▸). This growth model can explain the different efficiency of the B cap for thin and thick LaN films. At larger film thickness, the coating contains voids. Some of these voids reach up to the surface. If they are sufficiently large, they cannot be completely closed by the cap. At these points, the film can be attacked by oxygen and humidity. Once this process starts, the large volume increase due to hydroxide formation leads to a further destruction of neighbouring film areas, starting an avalanche-like degradation process. The proposed grain-boundary diffusion mechanism for La explains why, in this case, the formation of pores is suppressed, the B cap covers the entire film, and for larger film thicknesses no degradation is observed.

### 3.4. Nitridation of an already deposited lanthanum film   

**Figure 8:**
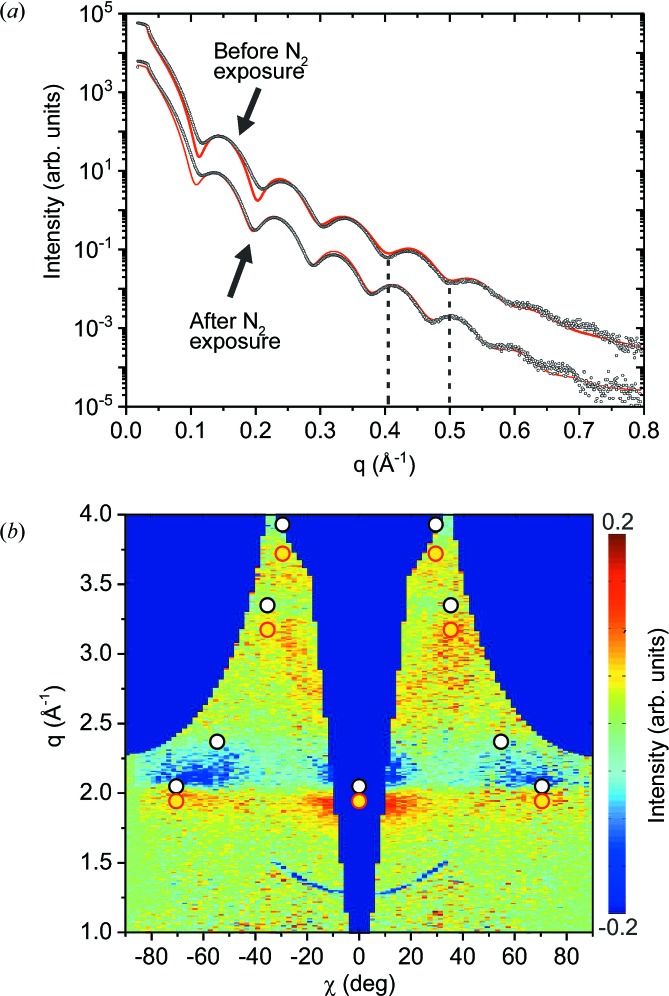
X-ray reflectivity curves measured before and after N_2_ exposure of a 2.7 nm thick lanthanum layer. Dots correspond to experimental data and fitted curves are presented as red lines. The fit parameters are summarized in Table 1 ▸. To highlight the change in the oscillation period, dashed lines indicate the positions of intensity minima before N_2_ exposure. (*b*) Difference between the XRD maps after and before N_2_ exposure. As a result of N_2_ exposure, the intensity decreases at the positions of [111] f.c.c. La (white dots) and increases at the positions of [111] ZB LaN (yellow dots).

The reflectance of La/B multilayer structures is significantly improved by a combination of non-reactive and reactive La deposition. This process, also called partial (delayed) La nitridation, results in an La/LaN/B stack (Kuznetsov *et al.*, 2015 ▸). It is expected that such a stacking sequence can also be produced by post-growth nitridation. Therefore, a thin lanthanum film was exposed for 10 min to a nitrogen flux of 4 sccm. Fig. 8 ▸(*a*) compares the angular reflectivity curves measured before and after N_2_ exposure. After nitridation, the oscillation minima are shifted to lower *q*, indicating a larger film thickness. A fit of the data revealed an increase in the film thickness of 0.35 nm.

To study the influence of nitridation on the crystalline structure, diffraction maps before and after nitridation were recorded. Fig. 8 ▸(*b*) shows the difference signal of the two maps, enhancing the relatively small intensity changes. The XRD intensity increases at the positions of [111] ZB LaN (yellow dots), while the intensity at the [111] f.c.c. La (white dots) decreases, indicating the transformation of the topmost atomic layers of La into LaN. For f.c.c. La, the volume per La atom is 37 Å^3^, and for ZB and WZ LaN it is about 42 Å^3^ (Ghezali *et al.*, 2008 ▸). Assuming that the volume increase is dominated by an expansion in the **z** direction where the atoms are free to move, a thickness increase of 0.35 nm would correspond to a nitride layer of 2.6 nm thickness. Interestingly, the texture of the La film is maintained even in the nitridized surface layer. This can be explained by the identical arrangement of La atoms in f.c.c. La and ZB LaN. The N atoms fill interstitial sites of the f.c.c. La lattice (see Fig. 5 ▸ *a*).

This indicates that the structure formation mechanism during post-growth nitridation is quite different from the formation mechanism during deposition. During deposition, the crystallites of both WZ and ZB phases are formed during a competitive nucleation and growth process. During post-growth nitridatiton, the crystalline phase forms *via* diffusion of nitrogen atoms in the already established f.c.c. lattice of the lanthanum atoms.

## 4. Summary and conclusions   

Using *in situ* synchrotron methods during non-reactive and reactive sputter deposition, it was possible to determine the crystalline structures and textures of highly reactive La and LaN thin films. For La, a dominant [111] texture of the expected NaCl structure was identified, coexisting with a weaker [100] texture. For LaN, in agreement with theoretical predictions, the observed diffraction peaks were explained by coexisting metastable WZ[001] and ZB[111] textures. The ZB phase was also found after post-growth nitridation of f.c.c. La. After initial deposition of an amorphous or nanocrystalline disordered layer, La and LaN thin films show a roughness transition related to texture formation. For La, this roughness transition is slightly delayed compared with the onset of texture formation. This is attributed to the greater mobility of the pure metal compared with the nitride, resulting in a different growth mechanism. For metal growth, grain-boundary diffusion plays an important role, while for nitride growth, shadowing effects dominate.

On the basis of the above observations, two possible explanations for the degradation of B-capped thick LaN films were identified. (i) After the onset of texture and facet formation, LaN is expected to form a voided structure as a result of shadowing effects. Some of these voids reach up to the sample surface and cannot be completely closed by the cap, thus allowing direct contact between the film and ambient conditions. (ii) The crystalline structure might contribute to the stability of the films. The observed WZ LaN structure has the same metal sublattice as La_2_O_3_, which might facilitate oxidation and subsequent hydridation. The post-growth nitridation of lanthanum, as a further development of the delayed nitridation process successfully employed to increase the reflectivity of thin LaN films, might solve the void problem since it maintains the dense structure of the initial film.

Direct experimental evidence for the proposed LaN void structure is challenging, since the bulk densities of WZ and ZB LaN are not yet known. Commonly, this question would be addressed by cross-sectional scanning and transmission electron microscopy studies. In the case of La and LaN, these methods are complicated by the high reactivity of the films. Atomic force and scanning electron microscopy measurements of the capped structures cannot resolve the expected large aspect ratio of the voids and are unlikely to detect local widely separated defects. Indirect evidence, however, is given by optical microscopy, which reveals that sample destruction proceeds *via* radially extending and widely scattered areas.

## Figures and Tables

**Table 1:** Real part correction δ of the refraction index, film thickness *d* and roughness σ used for the fit of the angular X-ray reflectivity measurements shown in Figs. 7 ▸(*b*) and 8 ▸(*a*)

Sample	δ × 10^6^	*d* (nm)	σ (nm)
Si	5.44		0.44
SiO_*x*_	4.48	0.18	0.44
La (thin)	10.01	6.37	0.31
La (thick)	10.08	29.7	0.53
LaN (thin)	8.67	2.76	0.31
LaN (thick, layer1/layer2)	9.43/10.67	0.88/28.42	0.31/0.51
La (thin, after nitridation)	10.13	6.73	0.35
